# Supplementary figures and images for: Macroscopic resting state model predicts theta burst stimulation response: A randomized trial
Source: PLoS Comput Biol. 2023 Mar 6;19(3):e1010958. doi: 10.1371/journal.pcbi.1010958 (PMC10019702; doi:10.1371/journal.pcbi.1010958)

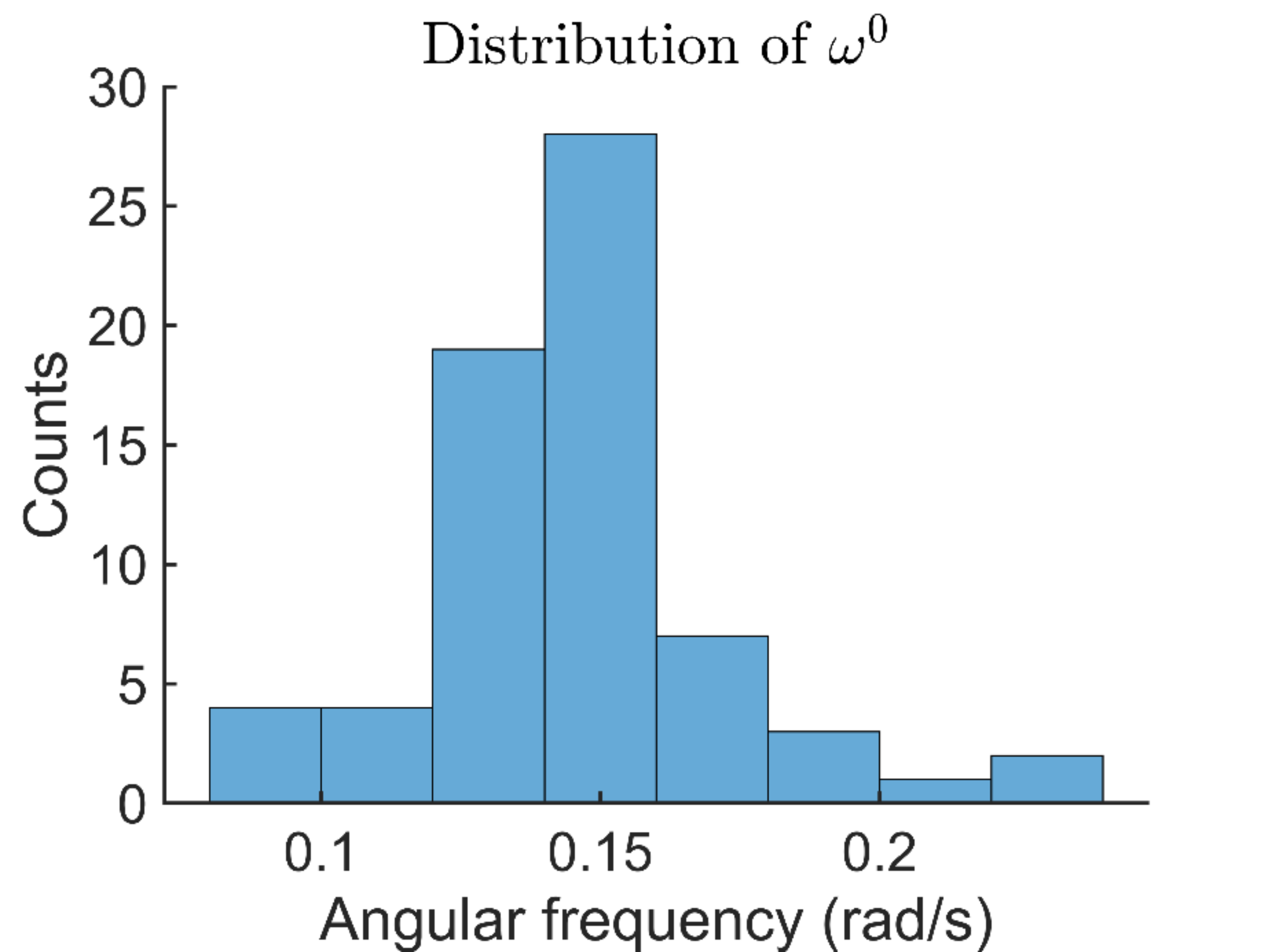

Supplement: S1 Fig — The peak of spectral density that was derived from Welch method was identified for each region at the individual level. The median value across the cohort was used as the group-representative value of ω°j in Eq 1. (TIF) [file pcbi.1010958.s001.tif]

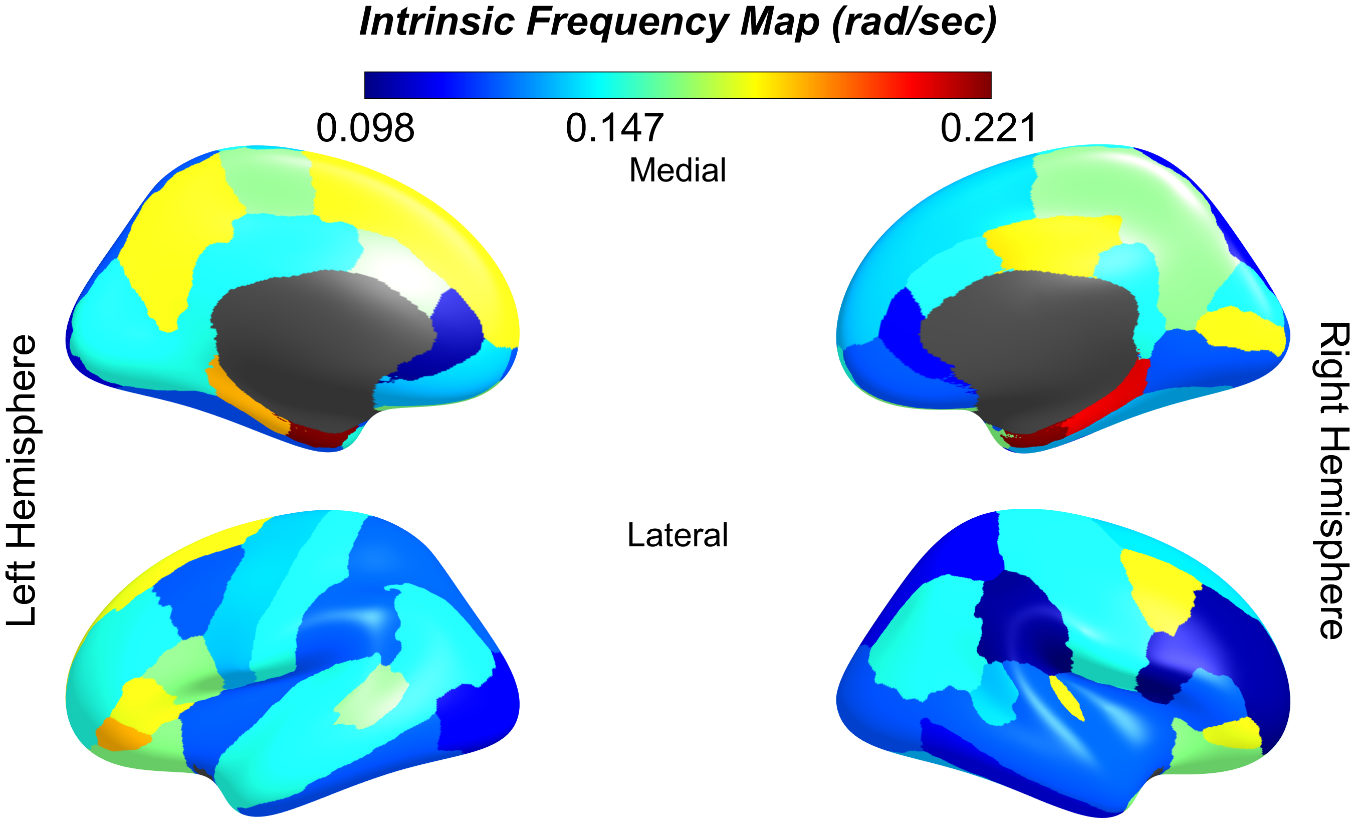

Supplement: S2 Fig — The intrinsic angular frequency is represented on the Desikan-Killany atlas using a color-coded scheme. Warmer colors indicate a higher intrinsic angular frequency whereas the cooler colors indicate a lower intrinsic angular frequency. (TIF) [file pcbi.1010958.s002.tif]

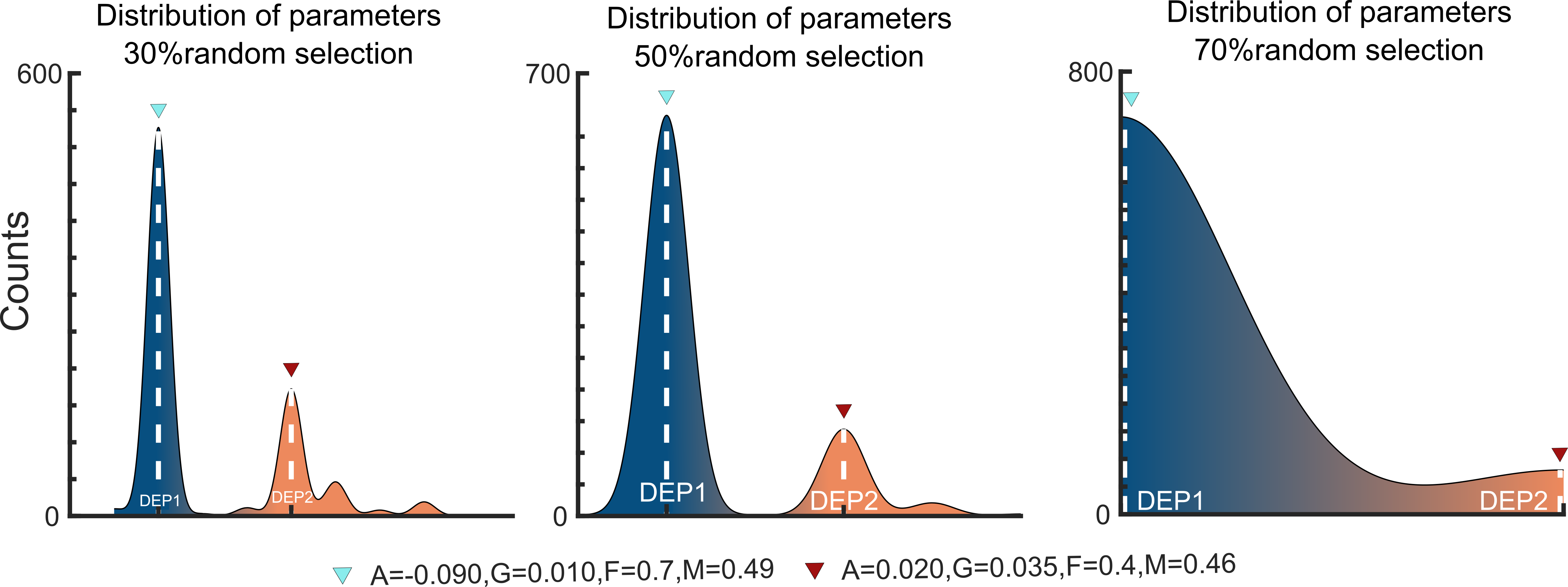

Supplement: S3 Fig — In Monte Carlo resampling, we randomly selected 30%, 50% or 70% (i.e., Monte Carlo threshold) of patients with 500 iterations per threshold. In all three threshold choices, we observed bimodal distributions with similar peak values (optimal parameter-sets), indicating that two subtypes were detectable at all three thresholds. (TIF) [file pcbi.1010958.s003.tif]

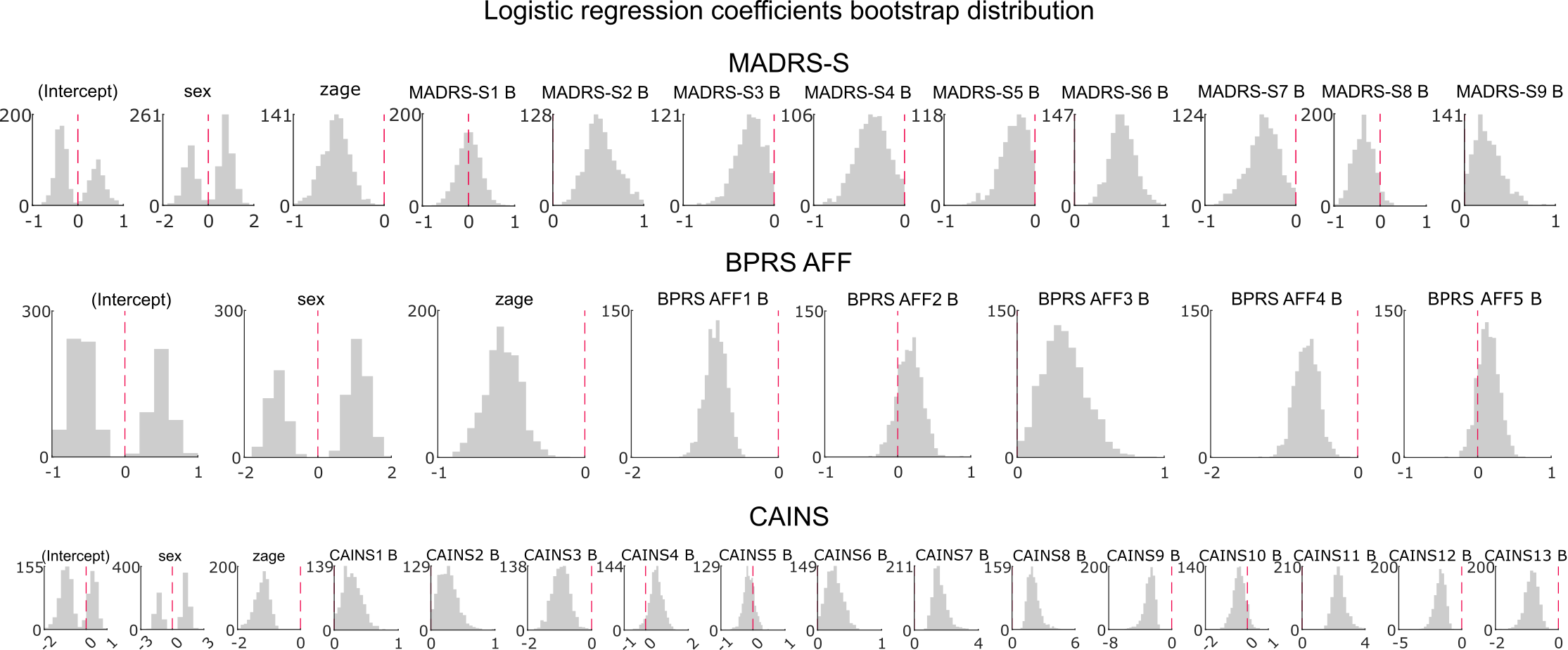

Supplement: S4 Fig — The distributions in each column represent a coefficient in the logistic regression model for MADRS-S (upper panel), BPRS AFF (middle panel) and CAINS (bottom panel) at baseline. The dashed red line depicts 0, which represents no association with the stratification of the depression cohort. At baseline, the bootstrap 95% confidence interval for MADRS-S2, MADRS-S4, MADRS-S6, MADRS-S7, BPRS AFF1, BPRS AFF3, BPRS AFF4, CAINS3, CAINS7, CAINS8, CAINS9, CAINS11, CAINS12 and CAINS13 did not include 0 at baseline, indicating a statistically significant association with the stratification of the depression cohort. (TIF) [file pcbi.1010958.s004.tif]

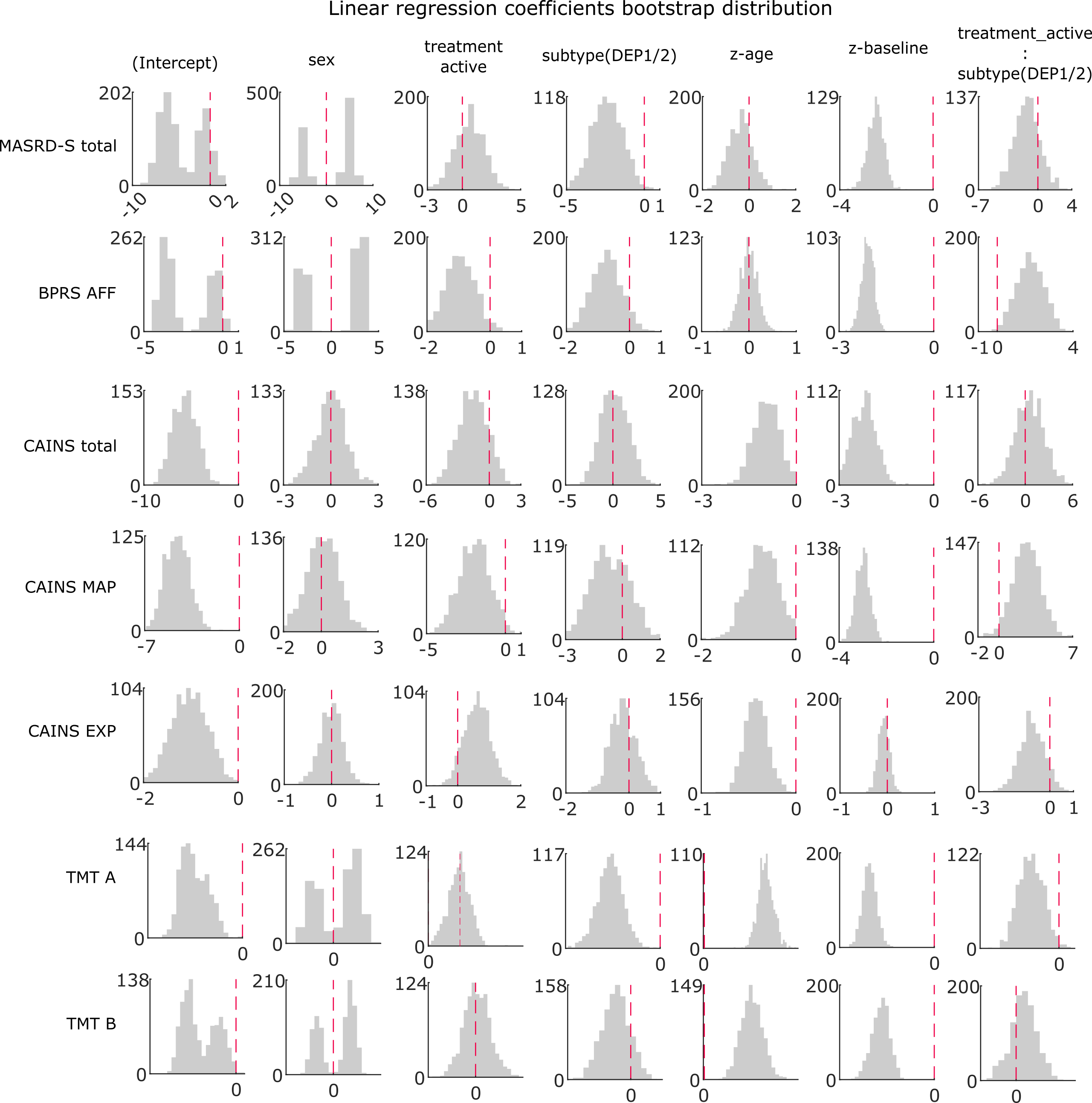

Supplement: S5 Fig — The distributions in each column represent a coefficient in the linear regression model where each row corresponds to a behavioral measure (i.e., outcome). The dashed red line depicts 0, which represents no association with the outcome. In this analysis, the coefficient of interest was the last column (treatment_active:subtype(DEP1/DEP2)). For BPRS AFF and CAINS MAP, the bootstrap 95% confidence interval for the treatment_active:subtype(DEP1/DEP2) did not include 0, indicating a statistically significant association with the outcome from baseline to follow-up. (TIF) [file pcbi.1010958.s005.tif]

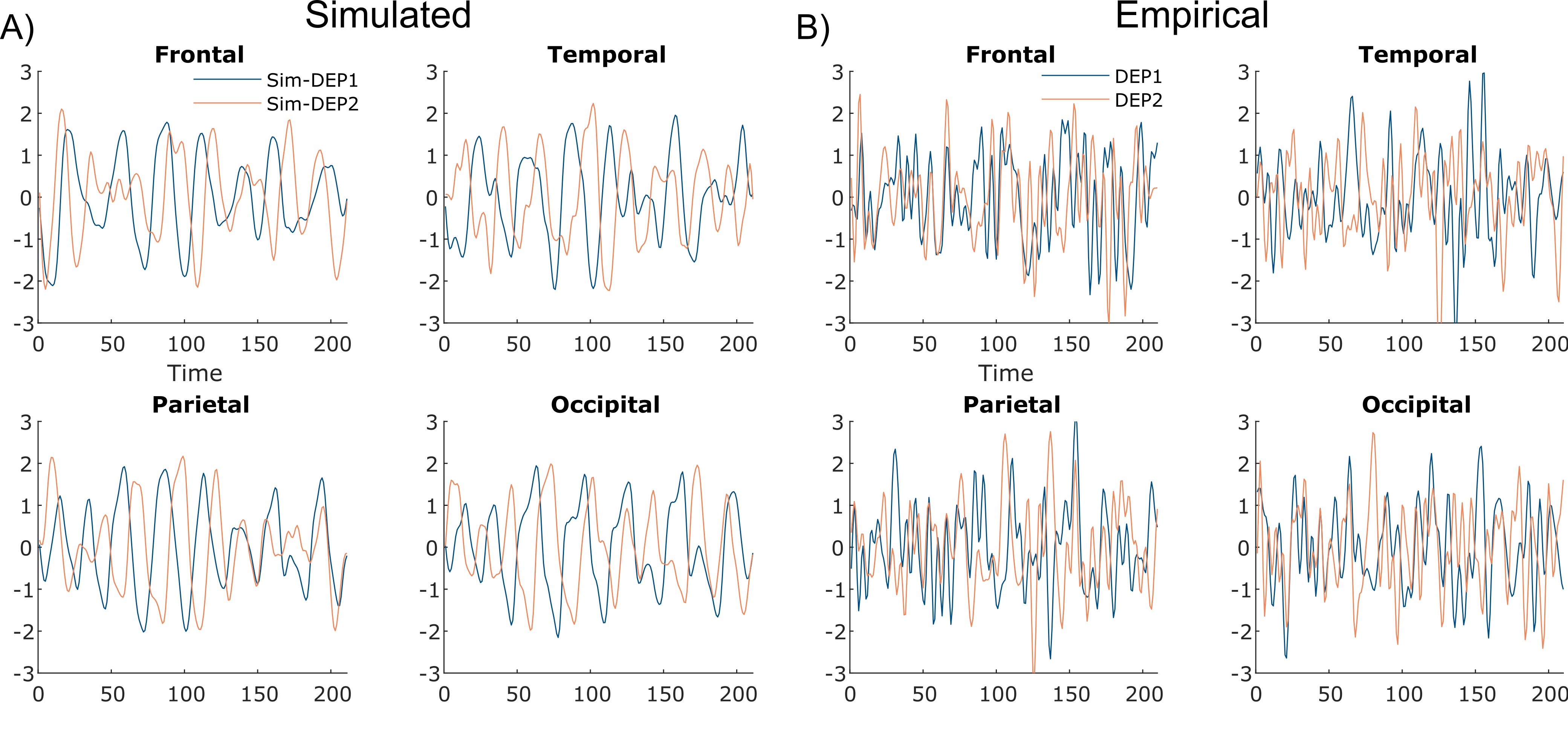

Supplement: S6 Fig — A) The simulated time-series for the two parameter-sets (i.e., DEP1: A = -0.090, G = 0.010, F = 0.7, M = 0.49 and DEP2: A = 0.020, G = 0.035, F = 0.4, M = 0.46) were averaged for all regions within each major brain lobe (i.e., Frontal, Temporal, Parietal and Occipital). B) The empirical BOLD time-series for each major brain lobe were averaged across individuals within each identified subtype. (TIF) [file pcbi.1010958.s006.tif]

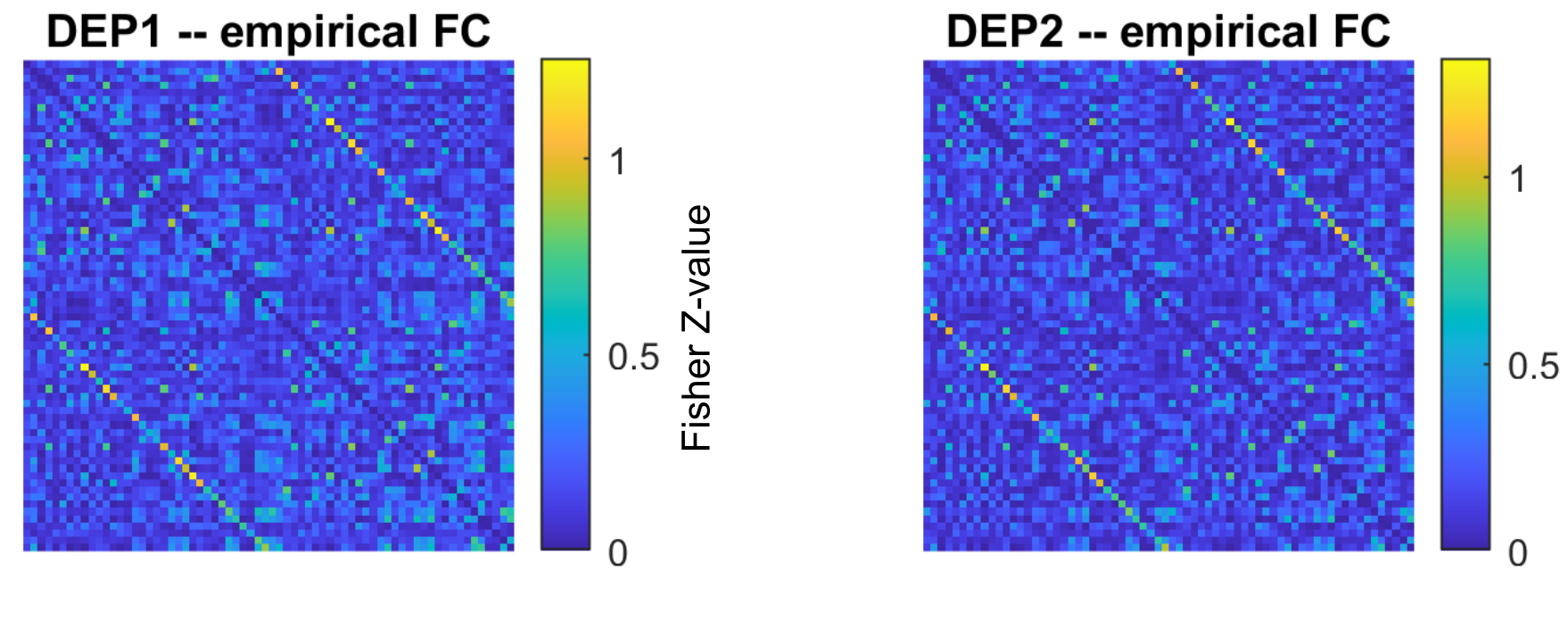

Supplement: S7 Fig — The empirical functional connectivity (FC) was derived from band-pass filtered BOLD signals. The resulting FCs were Fisher’s z-transformed, and any negative values were replaced with 0. (TIF) [file pcbi.1010958.s007.tif]

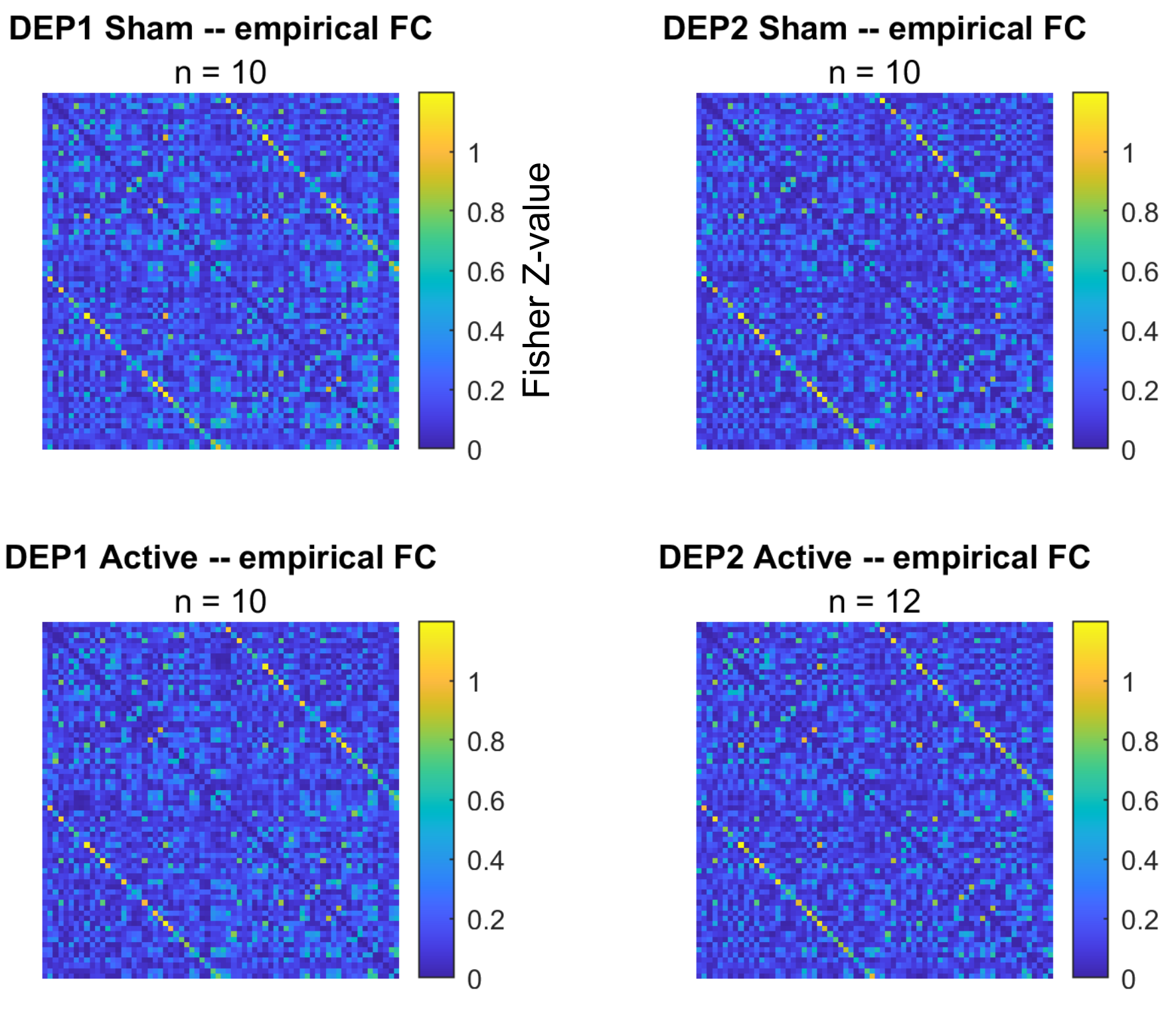

Supplement: S8 Fig — The band-pass filtered BOLD time-series were used to compute functional connectivity (FC) for depression subtypes and treatment groups. The resulting FCs were Fisher’s z-transformed, and any negative values were replaced with 0. The number of individuals contributing to each FC plot is written in the second line of the title. (TIF) [file pcbi.1010958.s008.tif]
