# Supplementary material for: Macroscopic resting state model predicts theta burst stimulation response: A randomized trial
Source: PLoS Comput Biol. 2023 Mar 6;19(3):e1010958. doi: 10.1371/journal.pcbi.1010958 (PMC10019702; doi:10.1371/journal.pcbi.1010958)
Supplement: Attachment — Submitted filename: Kaboodvand & Iravani et al_Response to reviewer_R2_final.docx [file pcbi.1010958.s010.docx]

Response to the reviewers

# Reviewer #1:

This is a very interesting and timely study which could be relevant for both basic research and clinical applications. Using whole-brain computational modelling, the authors were identified two depression subtypes exhibited different phenotypic behaviour.

Whereas the study design and rTMS protocol are in line with the standards of depression treatment, the computational modelling seems novel and requires some clarification.

The main concern regarding the results is whether the identified depression subtypes are genuine, and therefore cannot be explained by specific modelling settings.

**Reply:** Authors are delighted that the reviewer found some interesting aspects in our manuscript. The reviewer has raised an important point, whether the delineation of the cohort into two covert subgroups is a mere result of model settings. We would like to emphasize that the emergence of the two groups is rooted in the nature of the empirical data at question. Should a homogenous sample be fed in to the model, one set of optimal parameters will be achieved; as we previously demonstrated using similar analysis and modelling approach for healthy cohort (Iravani et al., 2021; Figure 1).

Also, in the reply to the following comments we indicated that the categorization of our cohort to two sub-groups was replicated when a new grid search range and different thresholds for the Monte Carlo permutation were used (please see reply to the **comment #9** and **comment #11**). Therefore, our whole-brain modelling approach is able to capture the potential heterogeneity in the sample.

## Technical notes:

### 1. Page 9, “Filtering was implemented using a discrete cosine transform windowing operation …”. Please specify the window length.

**Reply:** Band-pass filtering was performed as a part of the denoising step in CONN toolbox. In general CONN uses the same procedure as SPM which simply removes the out-of-band components in a DCT (discrete cosine transform) of the timeseries. The function *conn_filter.m* implements this (it uses a FFT of the even-symmetry-extended original timeseries; there are no additional default parameters there).

We now clearly wrote **page** **9**, **paragraph** **2**, **lines 7-9**, that we used the band-pass filtering function as implemented in the CONN toolbox:

*“Filtering was implemented by applying conn_filter.m function, implemented in Conn toolbox (Whitfield-Gabrieli and Nieto-Castanon 2012), using a discrete cosine transform windowing operation to minimize border effects.*”

Of note, the length of the analysis window when computing the discrete Fourier transform of time-series using the fast Fourier transform (FFT) algorithm was set to the size of each time-series signal. We wrote on **page 9**, **paragraph 2**, **lines** **9-11**:

*“The length of the window for computing the discrete cosine transform of time-series was set to the length of each time-series.”*

### 2. Page 10, Parameter “Cij” (structural connectivity matrix) in equation (1) is not defined/described explicitly. Please add the description.

**Reply:** Thank you for noting this. As mentioned by the reviewer, the Cij is the structural connectivity matrix which determines the level of connection between each pair of nodes.

On **page 10**, **paragraph 3** and **lines 4-6**, we now wrote**:**

*“In the Eq.1, Cij represents the level of structural connectivity between each pair of regions, which was global-wise normalized so that the structural connectivity weights of each region summed up to 1.”*

### 3. Page 10, “The variable represents the freely running frequency of region ”. This requires further explanation. How was the running frequency modelled, e.g., frequency range, specific distribution, etc.?

**Reply:** For every brain region j, the angular frequency ω_j_ represents the freely running frequency, which is modelled by the first order differential equation as follows:

$$\dot{\omega}_{j}={\omega^{0}}_{j}-F\omega_{j}+M\sum C_{ij}arg\left( z_{i} \right)$$

The above differential equation has a fixed-point attractor at ${{\left( \frac{1}{F} \right)\times(\omega}^{0}}_{j}+M\sum C_{ij}arg\left( z_{i} \right) )$. The intrinsic frequency, ${\omega^{0}}_{j}$, was estimated from the empirical BOLD signal of region j by applying the Welch method and finding the peak of power spectral density for each individual region. For every particular region, we took the median of estimated intrinsic frequency across the full cohort as the final ${\omega^{0}}_{j}$ value. The distribution of intrinsic angular frequencies has been shown in the **Fig S1**.

**Fig S1. The distribution of intrinsic angular frequencies.** The peak of spectral density that was derived from Welch method was identified for each region at the individual level. The median value across the cohort was used as the group-representative value of ${\omega^{0}}_{j}$ in the equation 1.

We added this new information to **page** **10**, **paragraph** **3** and **lines** **7-8**:

*“…, which was modeled by a first order differential equation, defined in the second line of Eq.1.”*

We also added the text below to **page** **10**, **paragraph** **3,** **lines 9-12**:

*“We estimated* ${\omega^{0}}_{j}$ *at the individual level by finding the peak of BOLD spectrum. Subsequently, the median value across the cohort was used as the group-representative value for* ${\omega^{0}}_{j}$ *in the range of 0.098–0.221 rad/sec,* ***Supplementary Fig. S1****.”*

### 4. Page 10, “0 denotes the intrinsic frequency at steady state for each region which was experimentally obtained and set to the median frequency of the regional BOLD signal”. Assuming that the power spectrum of a BOLD signal follows a power-law, what does it mean “intrinsic frequency”? To clarify this, could you plot the median frequency (averaged across patients) for each of 68 regions? If possible, could you plot these values on Desikan-Killany brain parcellation?

**Reply:** Thank you for raising this point. We referred to $\omega^{0}$ as the intrinsic angular frequency considering that it represents dominant resting state oscillations.

We followed the reviewer’s suggestion and illustrated the estimated intrinsic frequency values (median across all individuals) for all 68 regions of the Desikan-Killany parcellation (**Fig S2**).

***Fig S2. Estimated intrinsic angular frequency map****.* *The intrinsic angular frequency is color coded on the Desikan-Killany atlas. The warmer color represents higher intrinsic angular frequency whereas the cooler color denotes lower intrinsic angular frequency.*

We added this new figure to the supplementary materials.

Moreover, we added on **page** **10**, **paragraph** **3, lines 12-13**:

*“**Hence, the intrinsic angular frequencies represent the region-specific dominant oscillations during resting state,* ***Supplementary Fig. S2****.”*

### 5. Page 10, “Plus, an additive Gaussian noise (denoted as ηj, with the standard deviation of β = 0.02) was implemented as Wiener process to simulate random processes in brain”. What is the rationale behind β = 0.02? Have you tested other values?

**Reply:** The additive Gaussian noise models the dynamics of non-neuronal random processes and dose not directly change the attractor space. Consequently, systematically changing the standard deviation (β) was not necessarily of our potential interest.

In order for our search parameters to be aligned and remain in the same range with that of the previous research, we fixed the noise SD to β = 0.02, a value that has been previously used by us [(Iravani et al., 2021; Kaboodvand et al., 2019)](https://sciwheel.com/work/citation?ids=10474320,7267499&pre=&pre=&suf=&suf=&sa=0,0&dbf=0&dbf=0) as well as other researchers [(Deco et al., 2019, 2017; Senden et al., 2017)](https://sciwheel.com/work/citation?ids=4840565,7521653,3865159&pre=&pre=&pre=&suf=&suf=&suf=&sa=0,0,0&dbf=0&dbf=0&dbf=0).

We wrote on **page 10**, **paragraph 3**, **lines 20-21**:

*“In order for our search parameters to remain in the same range with that of the previous research* [(Iravani et al., 2021; Kaboodvand et al., 2019)](https://sciwheel.com/work/citation?ids=7267499,10474320&pre=&pre=&suf=&suf=&sa=0,0&dbf=0&dbf=0)*, we fixed the noise standard deviation to β = 0.02.”*

### 6. Page 10, “In order to fit the model to the fMRI data, we need to find an optimal working point, that is a combination of parameters (i.e., A, G, F and M) that maximizes the similarity between the simulated and empirical FC patterns and captures the intrinsic attractor dynamics of the brain”. Have you use any quantitative measure to describe how well the model captures the brain dynamics?

**Reply:** The objective of our model was mainly to reproduce the observed functional connectivity patterns of the human brain. To tackle this, we used a cost function that was based on the similarity of the simulated and empirical functional connectivity maps. Therefore, we used the similarity between the empirical and simulated functional connectivity data as the only quantitative measure of fitness.

We now clearly mentioned this matter on **page** **11**, **paragraph** **1**, **lines 4-5**:

*“Therefore, the similarity between the empirical and simulated FC was measured to quantify the goodness of fitting.”*

### 7. Currently, the signals plotted on Fig. 1A do not allow assessing the similarity between simulated and empirical signals. Could you provide an example of simulated and empirical signals from four brain regions (i.e., occipital, parietal, temporal, frontal) of a representative patient, as a supplementary figure?

**Reply:** Thank you for this suggestion. However, we have to emphasize that we did not fit the model to each patient, rather to a subset of patients that was randomly selected in each Monte Carlo iteration (for total of 1500 iterations). Yet, to follow the reviewer’s suggestion, we plotted the simulated BOLD signal for two parameter-sets, as well as the average of the empirical data for the subset of patients identified as DEP1 or DEP2, separately for each brain lobe.

***Fig S6. The simulated and empirical BOLD for major brain lobes. A****)* *The* *simulated time-series for the two parameter-sets (i.e., DEP1: A= -0.090, G = 0.010, F = 0.7, M = 0.49 and DEP2: A = 0.020, G = 0.035, F = 0.4, M = 0.46)* *were averaged for all regions within each major brain lobe (i.e., Frontal, Temporal, Parietal and Occipital).* ***B****) The empirical BOLD time-series for each major brain lobe were averaged across individuals within each identified subtype.*

We now added **Supplementary** **Fig. S6** to the supplementary materials and added the text below on **page 16**, **paragraph 1**, **lines 8-10:**

*“The simulated time-series associated with DEP1 and DEP2 as well as the empirical counterparts are illustrated in the* ***Supplementary Fig. S6****.”*

It has to be further clarified that the objective of our model is to reproduce the functional connectivity between regions based on a structural connectome rather than the time courses/signals of the regions per se. Please also see the reply to **Reviewer1’s comment #6**

We added on **page 16**, **paragraph 1**, **lines 10-12**:

*“**It should be pointed out that the objective of our model was to simulate the FC patterns similar to the FCs of the empirical data rather than simulating the individual time-series.”*

### 8. Page 10, “Accordingly, in a grid-search framework, we searched for parameter A from −0.044 to 0.044 … ”. This could potentially be a strong limitation of the study because the range is arbitrary defined and it seems different from that in Iravani et al., 2021. Could you explain the rationale for this range, and why is it different from your previous study?

**Reply**: Thank you for raising this point. The parameter A is expected to be around zero. The rationale is that the optimal brain function during resting-state requires that the macroscopic brain dynamics operate at the brink of Hopf bifurcation (i.e., A=0), where the system can spontaneously transition between oscillatory and non-oscillatory regimes (high metastability regime, please also see [(Deco et al., 2017)](https://sciwheel.com/work/citation?ids=4840565&pre=&suf=&sa=0&dbf=0). Therefore, we opted to narrow down our search range compared to our previous study, considering that the simulations were computationally heavy. Accordingly, reducing the number of the simulations in this study allowed us to increase the number of Monte Carlo simulations by two-folds compared to Iravani et al., 2021. However, with respect to the reviewer’s concern (please also see our reply to **Reviewer #1’s comment #9**), we repeated the grid-search with the larger ranges as it was suggested by the reviewer. Again, we were able to replicate the findings of two sub-cohorts with a better fit (the similarity of empirical FCs and simulated FCs improved from .238 and .240 to .339 and .333 for DEP1 and DEP2), albeit with slightly different parameters.

We now added the text below to **page 16**, **paragraph 1**, **lines 3-5**:

*“It is worth mentioning that given these two parameter-sets, the model produced regional BOLD-like signals with FC maps resembling the empirical FCs of depression subtypes (r_DEP1_ = 0.339 and r_DEP2_ = 0.333).”*

### 9. To make sure that the range of the parameters does not affect the results, could you redo the modelling on a coarser grid: A from −0.2 to 0.2 with a step-size of 0.01, G from 0.005 to 0.05 with a step-size of 0.005, F from 0 to 1 with a step-size of 0.1, and M from 0.1 to 0.5 with a step-size of 0.03.

**Reply**: We increased the spanned range of parameters as suggested by the reviewer. Hence, we searched A from -0.2 to 0.2 with a step-size of 0.01, G from 0.005 to 0.05 with a step-size of 0.005, F from 0 to 1 with a step-size of 0.1, and M from 0.1 to 0.5 with a step-size of 0.03. The new grid search resulted in 63,140 simulations that is significantly larger than the number of simulations in our pervious setup (i.e., 49,140).

The new search resulted in a slightly different optimal parameter sets, with a better fit to the empirical data. The method and result sections have been accordingly updated.

In summary, with the new parameter-sets we were again able to detect the two sub-cohorts. We found that the phenotypic behavior at baseline differed between the two detected subtypes. Moreover, we could replicate that the interaction between the two sub-groups and treatments showed differences in BPRS-AFF and CAINS-MAP. Furthermore, we also found that the individual items of MADRS-S, BPRS AFF and CAINS were improved in a specific subtype of depression cohort.

The summary of our findings can be found on **page 21**, **paragraph** **1,** **lines** **5-18**:

*“**We indicated that the two detected depression subtypes exhibited different phenotypic behavior related to certain affective and negative symptoms at baseline. We further found that the effect of treatment on the summative scores for affect subscale of BPRS, motivation and pleasure subscale of CAINS varied as a function of depression subtypes. Nevertheless, the effect on the summative scores were mostly driven by worsening of DEP2 by active treatment, potentially due to the heterogeneity of DEP2’s response to individual items of the measurements. We followed up on this observation by assessing the individual items of MADRS-S total, BPRS AFF and CAINS using nested ANOVA. We found that the behavior related to mood, initiative/lassitude, suicidality and motivation for work and school activities were indeed improved in the subtype with a lower metastable and synchronous pretreatment intrinsic brain activity (i.e., DEP2), despite worsening of the summative scores. Hence, our results suggested that the summative scores of phenotypic behaviors for depression is not well-suited for dissociating the depression subtypes.”*

Please also see the reply to **Reviewer #1’s comment #8**.

### 10. Page 11, “The correlation values were then converted to z-values …”. Why is it necessary?

**Reply**: Applying the Fisher’s z-transformation to the Pearson’s correlation values (i.e., the functional connectivity measures) is a common practice for any second-level analyses of functional connectivity measures. The distribution of Fisher-transformed correlation coefficients approximately follows a normal distribution enabling us to correlate the simulated and empirical FCs using another level of Pearson correlation.

We now wrote on **page 11**, **paragraph 2**, **lines 5-8:**

*“Of note, Fisher’s z-transformation was applied to both simulated and empirical FC measures with the aim of performing a subsequent correlation between the simulated and the empirical FCs as an index of the goodness of fit of the model.”*

### 11. Page 11, “The FC maps of half of the patients were randomly selected in each iteration of 1000 Monte Carlo resampling and used to find the optimal parameters for the whole-brain model”. This is an important point. Could you explain why is it important to select half of the patients (not 30% or 70%)? To further validate the modelling technique, could you redo the analysis by selecting (a) 30% and (b) 70% of the patients instead of half?

**Reply:** Thank you, we now repeated the Monte Carlo permutations with the additional thresholds of 30% and 70%, as recommended by the reviewer. We permuted 500 times for each threshold and fitted a normal kernel distribution to achieve the distributions associated with each threshold, **Supplementary Fig. S3**. Notably, we found identical parameter-sets with all three thresholds, 30%, 50% and 70%. Since the sub-cohorts were detected for all three choices of thresholds, we pooled all permutations in the revised version to achieve a total of 1500 permutations.

***Fig S3. Distribution of parameter-sets as a function of Monte Carlo threshold.*** *In Monte Carlo resampling, we randomly selected 30%, 50% or 70% (i.e., Monte Carlo threshold) of patients with 500 iterations per threshold. In all three choices of threshold, we observed bimodal distributions with similar peak values (optimal parameter-sets) and consequently the two subtypes were detectable in all three thresholds.*

We added **Supplementary Fig. S3** to the supplementary material and added the text below to **page 11**, **paragraph 2**, **lines 8-11:**

*“**The FC maps of a varying percentage of the patients (i.e., Monte Carol thresholds) were randomly selected over 1500 Monte Carlo iterations to find the optimal parameter-sets within the search intervals for the whole-brain model.* *We sampled the FC maps for 500 iterations per threshold (i.e., 30%, 50% and 70%) to remove the dependency of our results on the percentage of patients randomly selected in Monte Carlo resampling procedure.”*

Moreover, on **page 11**, **paragraph 2**, **lines 15-17**we added:

*“**We found bimodal distributions for all three Monte Carlo thresholds (****Supplementary Fig. S3****), and therefore we pooled all three distributions to yield a multi-threshold distribution with 1500 resampling.”*

We also updated the **Figure1b** distribution.

***Figure1B.*** *The distribution of parameter-sets obtained from 1500 Monte Carlo resamplings across three thresholds (i.e., 30%, 50% and 70%) indicated two peaks, representing two subtypes in the data, so-called DEP1 and DEP2.*

### 12. Page 11, “Applying the probabilistic nonparametric framework enabled us to find the hidden subtypes in the cohort corresponding to different potential local maxima in the parameter distributions”. This requires a detailed description. It remains unclear, what probabilistic nonparametric framework did you use?

**Reply:** We meant to refer to the Monte Carlo permutation tests that were used for finding the optimal working point, which pointed out two different sets of optimal parameters related to the two hidden subtypes in the cohort. Our Monte Carlo framework can be considered as a probabilistic approach given that every subject in each iteration is either included or excluded with the probability of 50% (probabilities of 30% and 70% were also included in the revised version, please also see reply to the **Reviewer#1’s comment #11**). Over many permutations (i.e., 1500), our approach produces a non-parametric bimodal distribution, where the two appeared peaks of the distribution were associated with the two different optimal parameter-sets, exhibiting heterogeneity in the brain dynamics.

We now clarified this issue on **page 11**, **paragraph 2**, **lines 17-20:**

*“Applying the probabilistic nonparametric framework (i.e., Monte Carlo permutation) enabled us to find the two hidden subtypes in our depression cohort, corresponding to the two different peaks in the parameters’ distribution.”*

### 13. Page 11, “Next, based on the similarity between each individual’s empirical and model-derived network measures (i.e., clustering coefficient and nodal strength), we assigned each subject to one of the models (i.e., optimal parameter sets)”. Have you use both measures: clustering coefficient and nodal strength? If so, how did you combine these measures together? It would be interesting to see a table (as supplementary material) where you report these values for each patient (with columns: Patient | Clustering coefficient (empirical, model-derived) | Nodal strength (empirical, model-derived)). It will give more insight regarding the variability of the measures.

**Reply:** Thank you for bringing this issue to our attention. After assessing our analysis codes, we realized that we only used the similarity between empirical and simulated FC maps. Unfortunately, we failed to write this accurately in the manuscript and apologize for the error in the text. We fixed this issue in the revised manuscript.

On **page 11**, **paragraph** **2**, **lines** **1-4**, we improved the text as follows:

*“Next, based on the similarity between each individual’s empirical FC and either of two model-derived FC maps (i.e., simulated FC_DEP1_ and simulated FC_DEP2_), we assigned each subject to one of the models (i.e., each model associated with one of the optimal parameter-sets identified within the search intervals).”*

We are now reporting the similarity of each individual empirical FC to model-derived FC maps for each parameter-set in the **Supplementary Table S1**.

### 14. Page 11, “Our whole-brain model at the optimal working point …”. Since definition of the optimal point is based on grid-search within a limited range and subsequent correlation between simulated and empirical connectomes, it is difficult to say that this point is truly optimal. It is very possible that there is another working point (associated with different model parameters) that provides slightly lower correlation between the empirical and simulated connectomes, but this point could be equally optimal, if the correlation is not significantly different from the maximum correlation. In your analysis, have you considered other working points with the correlation lower than maximum but above the chance level?

**Reply:** We agree with the reviewer that our optimization strategy can have limitations. Given that we are striving to solve a naturally ill-problem where there are probably many other working points that produce above chance level correlations.

We wrote on **page 23**, **paragraph 2**, **lines 1-3:**

*“…our grid-search spanned a limited interval of the parameter space and therefore the obtained optimal parameter-sets are contingent on our defined search scope.”*

Moreover, we are now clearing mentioning that the optimal working point is within the search interval of grid-search throughout the manuscript.

### 15. Page 14, “It's worth mentioning that given these two parameter-sets, the model produced regional BOLD-like signals with FC maps resembling the empirical FCs of depression subtypes (rDEP1 = 0.238 and rDEP2 = 0.240 )”. Could you plot these connectomes (i.e., FCs for DEP1 and DEP2) in a supplementary figure? Could you also report the correlation/similarity between these connectomes (i.e., FCs for DEP1 and DEP2)?

**Reply:** Thank you for this suggestion. We now plotted the connectome for DEP1 and DEP2.

***Fig S7. Functional connectome for depression subtypes.*** *The empirical functional connectivity (FC) was derived from band-pass filtered BOLD signals. The FCs were Fisher’s z-transformed and negative values were replaced with 0.*

This new figure was added to the supplementary material. Moreover, the similarity between these two functional connectomes was rho = 0.95.

We now wrote on **page** **16**, **paragraph** **1**, **lines** **12-13**:

“*The mean FCs for DEP1 and DEP2 are illustrated in the* ***Supplementary Fig. S7****. The similarity between the two subtypes’ FC was rho = 0.95.*”

### 16. Page 16, “Fig. 2”. The main concern here is that there is a large difference between "Sham:DEP2" and "Sham:DEP1, Active:DEP1, Active:DEP2". Could you please plot associated FCs in a supplementary figure? Could you also report the number of subjects in each group (i.e., Sham:DEP2, Sham:DEP1, Active:DEP1, Active:DEP2)?

**Reply:** We now plotted the functional connectome for each group in the **Supplementary Fig. S8**. Also, we are reporting “n” for each group in the figure.

***Fig S8. Functional connectome as a function of subtypes and treatment.*** *The band-pass filtered BOLD time-series were used to compute functional connectivity (FC) for depression subtypes and treatment groups. The FCs were Fisher’s z-transformed and negative values were replaced with 0. The number of individuals contributing to each FC plot is written in the second line of the title.*

Moreover, we wrote on **page** **18**, **paragraph** **2**, **lines 3-4**:

*“Hence, the cohort was grouped with four levels (i.e., Sham:DEP1, n=10; Sham:DEP1, n=10; Active:DEP1, n=10; Active:DEP2, n=12) for the LiM analysis.”*

Moreover, we added on **page 19**, **paragraph 1**, **lines 10-11**:

*“Moreover, The FC associated with each treatment group and depression subtype is depicted in the* ***Supplementary Fig. S8****.”*

### 17. “Data and Code availability statement”. I would like to ask you to clarify the Data and Code availability statement. It states that “The functional scans, behavioral data and code for analyzing are locally available upon reasonable request”. Could you please provide a reason why the code cannot be made public (upon manuscript acceptance)? Could you also provide information regarding the restrictions associated with distribution of the functional scans and behavioural data?

**Reply:** Thank you for bringing this matter to our attention. All the authors are in favor of open science framework and we happily shared our modeling codes on the public repository. The codes are publicly accessible at: ‘https://github.com/Behzad-Iravani/WBM-TMS-and-Depression-‘

Yet, we are not able to publicly share the functional and behavioral data due to the institutional and ethical concerns.

We updated our Data and Code availability statements as follows:

*“The structural data used for creating the computational models is openly available at* [*http://www.humanconnectomeproject.org/data/*](http://www.humanconnectomeproject.org/data/)*. The functional scans and behavioral data cannot be publicly shared due to the institutional and ethical constraints; however, are locally available upon reasonable request. The code for the whole-brain modeling is publicly available at* [*https://github.com/Behzad-Iravani/WBM-TMS-and-Depression-*](https://github.com/Behzad-Iravani/WBM-TMS-and-Depression-)*.*”

# Reviewer #2:

### 1. It seems that the results should be reframed according to differential MDD subtype response to placebo/sham rTMS given that this was the group difference driving the interaction between subtype and outcome. This perspective is further supported by the lack of clinical effect (no sig difference for active vs. sham rTMS treatment).

**Reply:** Authors thank the reviewer for the constructive comments. We agree with the reviewer that this is an important aspect to highlight in the manuscript.

Based on Reviewer #1’s comments we repeated our modeling with a modified setup (please see the reply to **Reviewer #1’s comment #9** and **comment #11** ). Accordingly, we were able to replicate the finding of two covert subtypes with the new grid-search and different thresholds of Monte Carlo permutations. The new parameters are slightly different than those we reported in the original manuscript, and they are leading to a higher similarity between the model output and the empirical data. The similarity improved from .238 and .240 to .339 and .333 for DEP1 and DEP2 (please also see the reply to **Reviewer #1’s comment #8**).

With our new parameters we now found that active treatment in DEP2 was driving the observed effect. However, the effect was in an opposite direction. It should be noted that in this analysis the linear regression was performed on the summative scores of depression (i.e., MADRS-S), affective psychiatric symptoms (i.e., BPRS AFF) and motivation and pleasure subscales of negative symptoms (i.e., CAINS MAP). We hypothesized that heterogeneity in response to the individual items was possibly a constraint leading to no effect or opposite direction effect. Therefore, considering that we found a modulation of behavior, regardless of its direction, as a function of depression subtype and treatment, we followed up on the linear regression analysis by performing a nested ANOVA on the individual items of MADRS-S, BPRS AFF and CAINS. Indeed, this was an exploratory analysis, and we now clearly mentioned this limitation in the manuscript (please also see the reply to the **Reviewer #2’s comment #2**). Our nested ANOVA resulted in finding an improvement in MADRS-S1, MADRS-S6, BPRS AFF3, BPRS AFF4 and CIANS5 for DEP2 compared to the DEP1, while controlled for the sham treatment.

We revised the whole result section accordingly.

### 2. The item by item analysis on the symptom scales should be highlighted as exploratory and uncorrected for multiple comparisons which is a major concern for interpreting these findings. The item 8 effects are thus very unlikely to replicate which should be explained clearly in the manuscript.In fact, the result should not be interpreted given the statistical problem and, instead, mentioned only as a preliminary finding that needs to be replicated before it can be confirmed.

**Reply:** We agree with the reviewer that our results need to be interpreted with caution. However, we highlight that the main objective of our manuscript was to bring up the application of whole-brain modeling for stratification of depression cohort to the attention of our readers. We do agree that more future studies are required to validate such delineation of depression cohort before it finds its way into clinical practice.

We now wrote on **page 23**, **paragraph 2**, **lines 3-6:**

*“Moreover, the small sample size bounded our model prediction and the statistical analysis. Furthermore, our nested ANOVA analysis was an exploratory analysis; therefore, our results should be considered as preliminary findings and have to be validated in an independent sample.”*

### 3. It should also be mentioned that the subtypes themselves need to be replicated in independent samples to confirm their validity for understanding heterogeneity in clinical response to rTMS at this site.

**Reply:** Thanks for bringing this matter to our attention.

We now wrote on **page** **24**, **paragraph** **1**, **lines** **5-7**:

“Nevertheless, it should be noted that this type of stratification of the depression cohort is novel and has to be further validated in independent samples before finding its way into the clinical practices.”

## Minor issues

### 1. The Cole et al study did not confirm mechanism (not even pre post fMRI) so the claim that effects happened “via accumulating nonlinear strengthening of synapses” should be removed.

**Reply:** Thank you. The text has been amended by removing that segment.

### 2. Evidence that the foot MT is better than hand for deep targets should be provided given the claim.

**Reply:** As the corresponding motor cortex lies as the same depth as our target region, this ensures high enough stimulation intensity for reaching our prefrontal target. See also [(Downar et al., 2012)](https://sciwheel.com/work/citation?ids=14245154&pre=&suf=&sa=0&dbf=0)

We added on **page 5, paragraph 2, lines 9-11:**

*“The corresponding motor cortex lies at similar depth as our target region which ensured that our stimulation intensity was strong enough to reach the dmPFC* [(Downar et al., 2012)](https://sciwheel.com/work/citation?ids=14245154&pre=&suf=&sa=0&dbf=0)*.”*

### 3. Evidence that this coil reaches thee dmPFC target should be provided.

**Reply:** The most direct evidence is provided by Hayward et al. 2007 showing that dmPFC rTMS modulates regional cerebral blood flow in our target region [(Hayward et al., 2007)](https://sciwheel.com/work/citation?ids=3042550&pre=&suf=&sa=0&dbf=0). Further, indirect evidence is that we determined the motor threshold by stimulating the medial primary motor cortex and observed muscle contractions in extensor hallucis longus, a cortical area situated at similar depth as our prefrontal stimulation target.

We wrote on **page 5**, **paragraph 2**, **lines 3-5:**

*“It has previously been reported that applying rTMS over the medial frontal cortex, using the same coil type increases the cerebral blood flow in the dmPFC* [(Hayward et al., 2007)](https://sciwheel.com/work/citation?ids=3042550&pre=&suf=&sa=0&dbf=0)*.”*

Please also see the reply to the **Reviewer #2’s minor comment #2**

### 4. If the iTBS protocol exactly matches that of the original published (Huang et al.), a reference should be provided. If not, an explanation should be provided.

**Reply:** The iTBS protocol matches that of Huang et al. 2005. In our study, iTBS is then applied for each hemisphere and in two sessions as described in Bakker 2015, that we refer to.

We added the reference to Huang et al. 2005 in **the first line** of the “**Treatment**” section on **page 5**:

“*We applied the neuro-navigated iTBS* [(Huang et al., 2005)](https://sciwheel.com/work/citation?ids=334598&pre=&suf=&sa=0&dbf=0) *to dmPFC …”*

### 5. At 1st mention of the Magventure TMS coil, please provide a model for that coil as they sell many different models.

**Reply:** The coil model was Cool-DB80 A/P.

We added the additional information to **page 5**, **paragraph 2**, **line 3:**

*“…using the stimulator MagPro X100 and the MagVenture coil (Cool-DB80 A/P).”*

### 6. “To achieve bilateral dmPFC stimulation…we reversed the direction of current” This statement is not supported by the literature. Reversing current does not hop the induced field to the other hemisphere as a rule.

**Reply:** Our study followed Downar et al. 2012 who used the same approach [(Downar et al., 2012)](https://sciwheel.com/work/citation?ids=14245154&pre=&suf=&sa=0&dbf=0). This procedure was suggested to result in contralateral motor response in lower extremities when applied over the motor cortex. However, we agree with the reviewer regarding the importance of follow-up studies and further investigation to support this notion.

We added the reference to Downar et al. 2012 to **page 6**, **paragraph 1**, **lines 1-2:**

*“Reversing the direction of current has been previously shown to induce contralateral motor response in lower extremities, when applied over the motor cortex* [(Downar et al., 2012)](https://sciwheel.com/work/citation?ids=14245154&pre=&suf=&sa=0&dbf=0)*.”*

### 7. “reaching the predetermined target intensity” Does this mean that the targeted stim level was not reached until quite late in the treatment for some patients? This factor should be accounted for in the analyses since the effective dose would have been different for each patient. Same with the confound of patients who “did not meet these criteria” (presumably never reached the targeted stim level). How often did this happen and what effect did it have on the outcomes?

**Reply**: This is more fully described in [(Bodén et al., 2021)](https://sciwheel.com/work/citation?ids=12137913&pre=&suf=&sa=0&dbf=0) with the number of patients receiving full treatment reported. Since additional treatment days were added, we believe that we compensated for the low intensity treatment providing comparable effective dosage. Importantly, sham and active treatment did not differ in terms of the number of treatment days.

On **page 6**, **paragraph 1**, **lines 5-7**, we added:

*“Therefore, any potential low intensity treatment was compensated with the extra days providing comparable effective dosage across individuals.”*

### 8. “identical…protocol…sham” Does this mean that some patients also did not tolerate sham stimulation at the intended target? How did this affect outcomes?

**Reply:** This is accounted for in the answer above, with the total number of treatment days not differing between active and sham conditions. And yes, as the sham condition included TENS-stimulation of the medial forehead, which also can be painful, some patients had difficulties in tolerating sham stimulation at the intended target strength right away.

We wrote on **page 6**, **paragraph 4**, **lines 10-11:**

*“It is worth to further noting that there was no difference in the number of treatment days across active and sham groups* [(Bodén et al., 2021)](https://sciwheel.com/work/citation?ids=12137913&pre=&suf=&sa=0&dbf=0)*. “*

### 9. How well did sham work? This is important to determine whether the patients were indeed blind to their condition. This should be quantified in any sham/active comparison study.

**Reply:** We would like to refer to [(Bodén et al., 2021)](https://sciwheel.com/work/citation?ids=12137913&pre=&suf=&sa=0&dbf=0), where the blinding procedure is described. Notably, as reported in Boden et al. 2021, there was no difference in correct speculation of treatment allocation across the treatment groups.

Please also see **page 4**, **paragraph 3**, **lines 1-3**:

*“This study uses data from a randomized parallel double-blind sham-controlled trial which was pre-registered at the U.S. National Library of Medicine (i.e., ClinicalTrials.gov), with the registration ID of NCT02905604.”*

### 10. For the TMT, what version was used and what are the psychometric properties?

**Reply:** The TMT was a computerized version of the task as described in Reitan, 1958, Perceptual and Motor Skills, 8, 271-276.

Please see **page 8**, **paragraph 2**, **lines 2-3:**

*“TMT consists of two tasks with an increasing level of difficulty from task A to task B* [(Reitan, 1955)](https://sciwheel.com/work/citation?ids=9115160&pre=&suf=&sa=0&dbf=0)*.”*

### 11. Why rely on structural measures to capture ‘dynamics’ when actual time resolved fMRI data are available?

**Reply:** We made an attempt to integrate multi-modal imaging and bridge the gaps between the structural and functional data with the help of whole-brain computational modelling. Combining the two neuroimaging modalities alongside modelling the dynamics of brain regions provided us a unique opportunity to study the brain in a holistic manner. Assessing just the actual time resolved fMRI agnostic to the structural connectome and local brain dynamics does not provide enough information to perform similar type of analysis and delineate the cohort into the covert subtypes.

We added the sentence below to **page 23**, **paragraph 3**, **lines 2-3:**

*“Our approach provided a mathematical platform to efficiently combine the tractography and functional imaging data, while taking the nonlinear dynamics of the brain into account.”*

### 12. Why not use individual patient FC to guide targeting since they were collected?

**Reply:** This is indeed a great suggestion. However, it might be more advantageous for finer granulated methods of stimulation (e.g., Deep Brain Stimulation). We believe that using the structural imaging-based (T1-weighted MRI) guiding of the stimulation at the mesoscale, as occurs in TMS, is sufficient. Moreover, there is evidence showing that our procedure effectively modulates cerebral blood flow in the dmPFC (for more details please see [(Hayward et al., 2007)](https://sciwheel.com/work/citation?ids=3042550&pre=&suf=&sa=0&dbf=0)).

# Reference:

[Bodén, R., Bengtsson, J., Thörnblom, E., Struckmann, W., Persson, J., 2021. Dorsomedial prefrontal theta burst stimulation to treat anhedonia, avolition, and blunted affect in schizophrenia or depression - a randomized controlled trial. J. Affect. Disord. 290, 308–315. doi:10.1016/j.jad.2021.04.053](https://sciwheel.com/work/bibliography/12137913)

[Deco, G., Cruzat, J., Cabral, J., Tagliazucchi, E., Laufs, H., Logothetis, N.K., Kringelbach, M.L., 2019. Awakening: Predicting external stimulation to force transitions between different brain states. Proc Natl Acad Sci USA 116, 18088–18097. doi:10.1073/pnas.1905534116](https://sciwheel.com/work/bibliography/7521653)

[Deco, G., Kringelbach, M.L., Jirsa, V.K., Ritter, P., 2017. The dynamics of resting fluctuations in the brain: metastability and its dynamical cortical core. Sci. Rep. 7, 3095. doi:10.1038/s41598-017-03073-5](https://sciwheel.com/work/bibliography/4840565)

[Downar, J., Sankar, A., Giacobbe, P., Woodside, B., Colton, P., 2012. Unanticipated Rapid Remission of Refractory Bulimia Nervosa, during High-Dose Repetitive Transcranial Magnetic Stimulation of the Dorsomedial Prefrontal Cortex: A Case Report. Front. Psychiatry 3, 30. doi:10.3389/fpsyt.2012.00030](https://sciwheel.com/work/bibliography/14245154)

[Hayward, G., Mehta, M.A., Harmer, C., Spinks, T.J., Grasby, P.M., Goodwin, G.M., 2007. Exploring the physiological effects of double-cone coil TMS over the medial frontal cortex on the anterior cingulate cortex: an H2(15)O PET study. Eur. J. Neurosci. 25, 2224–2233. doi:10.1111/j.1460-9568.2007.05430.x](https://sciwheel.com/work/bibliography/3042550)

[Huang, Y.-Z., Edwards, M.J., Rounis, E., Bhatia, K.P., Rothwell, J.C., 2005. Theta burst stimulation of the human motor cortex. Neuron 45, 201–206. doi:10.1016/j.neuron.2004.12.033](https://sciwheel.com/work/bibliography/334598)

[Iravani, B., Arshamian, A., Fransson, P., Kaboodvand, N., 2021. Whole-brain modelling of resting state fMRI differentiates ADHD subtypes and facilitates stratified neuro-stimulation therapy. Neuroimage 231, 117844. doi:10.1016/j.neuroimage.2021.117844](https://sciwheel.com/work/bibliography/10474320)

[Kaboodvand, N., van den Heuvel, M.P., Fransson, P., 2019. Adaptive frequency-based modeling of whole-brain oscillations: Predicting regional vulnerability and hazardousness rates. Network Neuroscience 3.](https://sciwheel.com/work/bibliography/7267499)

[Reitan, R.M., 1955. The relation of the trail making test to organic brain damage. J. Consult. Psychol. 19, 393–394. doi:10.1037/h0044509](https://sciwheel.com/work/bibliography/9115160)

[Senden, M., Reuter, N., van den Heuvel, M.P., Goebel, R., Deco, G., 2017. Cortical rich club regions can organize state-dependent functional network formation by engaging in oscillatory behavior. Neuroimage 146, 561–574. doi:10.1016/j.neuroimage.2016.10.044](https://sciwheel.com/work/bibliography/3865159)

[Whitfield-Gabrieli, S., Nieto-Castanon, A., 2012. Conn: a functional connectivity toolbox for correlated and anticorrelated brain networks. Brain Connect. 2, 125–141. doi:10.1089/brain.2012.0073](https://sciwheel.com/work/bibliography/24512)
